# Large-Scale Genomic Analysis Suggests a Neutral Punctuated Dynamics of Transposable Elements in Bacterial Genomes

**DOI:** 10.1371/journal.pcbi.1003680

**Published:** 2014-06-26

**Authors:** Jaime Iranzo, Manuel J. Gómez, Francisco J. López de Saro, Susanna Manrubia

**Affiliations:** 1 Centro de Astrobiología (CAB), INTA-CSIC, Torrejón de Ardoz, Madrid, Spain; 2 Grupo Interdisciplinar de Sistemas Complejos (GISC), Madrid, Spain; University of Washington, United States of America

## Abstract

Insertion sequences (IS) are the simplest and most abundant form of transposable DNA found in bacterial genomes. When present in multiple copies, it is thought that they can promote genomic plasticity and genetic exchange, thus being a major force of evolutionary change. The main processes that determine IS content in genomes are, though, a matter of debate. In this work, we take advantage of the large amount of genomic data currently available and study the abundance distributions of 33 IS families in 1811 bacterial chromosomes. This allows us to test simple models of IS dynamics and estimate their key parameters by means of a maximum likelihood approach. We evaluate the roles played by duplication, lateral gene transfer, deletion and purifying selection. We find that the observed IS abundances are compatible with a neutral scenario where IS proliferation is controlled by deletions instead of purifying selection. Even if there may be some cases driven by selection, neutral behavior dominates over large evolutionary scales. According to this view, IS and hosts tend to coexist in a dynamic equilibrium state for most of the time. Our approach also allows for a detection of recent IS expansions, and supports the hypothesis that rapid expansions constitute transient events—punctuations—during which the state of coexistence of IS and host becomes perturbated.

## Author Summary

Insertion sequences (IS) are mobile genetic elements found in most prokaryotic genomes. They are able to autonomously change position and proliferate in chromosomes. The nature of the coevolutionary dynamics of IS with the genome that hosts them is a matter of debate: Do IS proliferate to the point of causing the extinction of the host? Is it possible that IS and hosts stably coexist? Can environmental perturbations cause IS expansions? What is the role of selection in controlling IS copy number? In this study, we have analysed abundance patterns of IS families to test two different evolutionary hypotheses: in the first one IS evolve neutrally, while in the second case they are affected by selection. Our results indicate that, most of the time, IS and their hosts coexist stably in a neutral scenario where the proliferation of IS through duplications and lateral gene transfer is balanced by regular deletions. Occasionally, though, this balance may be disrupted, causing temporary explosions of IS abundance.

## Introduction

Transposable elements (TE) are pieces of DNA that encode the enzymatic capability to change location and proliferate within the host genome through a process called transposition. They are widely distributed in prokaryotes and eukaryotes, and in some cases they constitute substantial fractions of the genome [1]. Due to their relative autonomy, proliferative ability, and apparent lack of a useful function, they were considered for some time a paradigm of selfish DNA, i.e. a molecular parasite that proliferates at the cost of the genome it “infects” [2], [3]. Nowadays, the relationship between TE and host genomes is known to be much more complex. Particular TE insertions may be beneficial for the host, for instance by inactivating genes whose expression is no longer required [4], [5], acting as a vehicle for the exchange of useful genes, or facilitating adaptation to fast environmental changes [1], [6], [7]. Even if TE did not play any beneficial role, hosts often possess regulatory mechanisms that keep TE under control and minimize the risk of possibly deleterious insertions [8], [9]. Because of their ability to promote recombination, TE are key contributors to the plasticity of genomes [10], [11]. Hence, understanding the dynamics of TE in different organisms is relevant to the comprehension of genome architectures.

Insertion sequences (IS) are the simplest form of TE, as they often code for only one gene responsible for their mobility machinery (the transposase gene) [9]. IS first enter host genomes through lateral gene transfer (LGT) and they can increase their copy number via transposition. The broad diversity of effects that IS exert on their hosts has turned the fate of this relationship —long-term coexistence or eventual extinction of the host due to IS proliferation—, into a matter of debate [12]. Moreover, relatively recent cases of rapid IS expansions in bacterial genomes, which have been attributed to episodes of host restriction and environmental change, raise additional questions on the causes and nature of such IS expansions [11], [13], [14]. As of today, the mechanisms by which environmental perturbations cause IS expansions, the role played by selection in controlling IS copy number, or the significance of decreases in host population sizes in the expansion of IS are mostly unsolved issues. Even more interestingly, could IS expansions represent transitory punctuations with a relevant role on host evolution? [15]–[17]. A better understanding of the evolutionary forces that control the IS dynamics is required in order to shed light on all these questions [18].

The first works aiming at analyzing TE dynamics date back to the decade of 1980 [19]–[22]. Inspired by the idea that TE are selfish elements, they depicted a scenario where TE spontaneously tend to proliferate and either host regulatory mechanisms or purifying selection keep TE numbers under control [21], [23]. Due to the limited data on TE abundance and distribution available at that time, those works either remained mostly theoretical or mainly addressed eukaryotic TE [24]. In recent years, however, the ever increasing number of sequenced genomes has provided us with an unprecedented amount of data on the abundance and distribution of prokaryotic TE. This has permitted the evaluation of a series of hypotheses concerning IS dynamics [14], [25]–[28]. In particular, a high homology of IS copies within genomes has been reported [25] and interpreted on the basis of a fast proliferation dynamics following the arrival of an IS element, ultimately leading to the extinction of the host. This view has been challenged [27] by the large proportion of IS remnants in *Wolbachia* genomes, implying that IS proliferation does not necessarily lead to extinction. Statistical approaches directed at identifying the causes behind IS abundance have found that it correlates with genome size but not with LGT rate, host pathogenicity or lifestyle [26]. Estimations of the fitness cost of IS elements by comparing a simple model with the genomic data available for the IS5 family [28] have found that the fitness cost is small enough to assume that, in practice, IS may be neutral or almost neutral for the host genome.

In this study, we take advantage of the large amount of genomic data currently available and analyze the abundance distributions of 33 IS families in 1811 bacterial chromosomes. This allows us to test and compare two simple models of IS spreading, namely a neutral model and a model with purifying selection, which are introduced in the next section. By fitting those models to the genomic data we obtain estimates for the proliferation, loss and LGT rates, as well as the fitness cost associated to an IS copy. The joint evaluation of such estimates and the original data allows us to disentangle the general forces that control IS dynamics in the long-term and explore the possibility that IS and hosts coexist in an equilibrium state punctuated by transient episodes of IS proliferation.

## Results

### Models of IS spreading and loss

The models here used are aimed at capturing the main mechanisms that are responsible for the proliferation, spreading and loss of IS within and among genomes. We first introduce a neutral model that takes into account the following key processes: (a) the IS ability to proliferate, (b) IS deletion, and (c) IS incorporation through lateral gene transfer (LGT). As an alternative to this neutral model, we also consider the case of IS entailing a fitness cost. The processes of proliferation, deletion, and LGT, complemented with a fitness cost that is proportional to the IS copy number, define a model of IS dynamics with selection.

**Figure 1:**
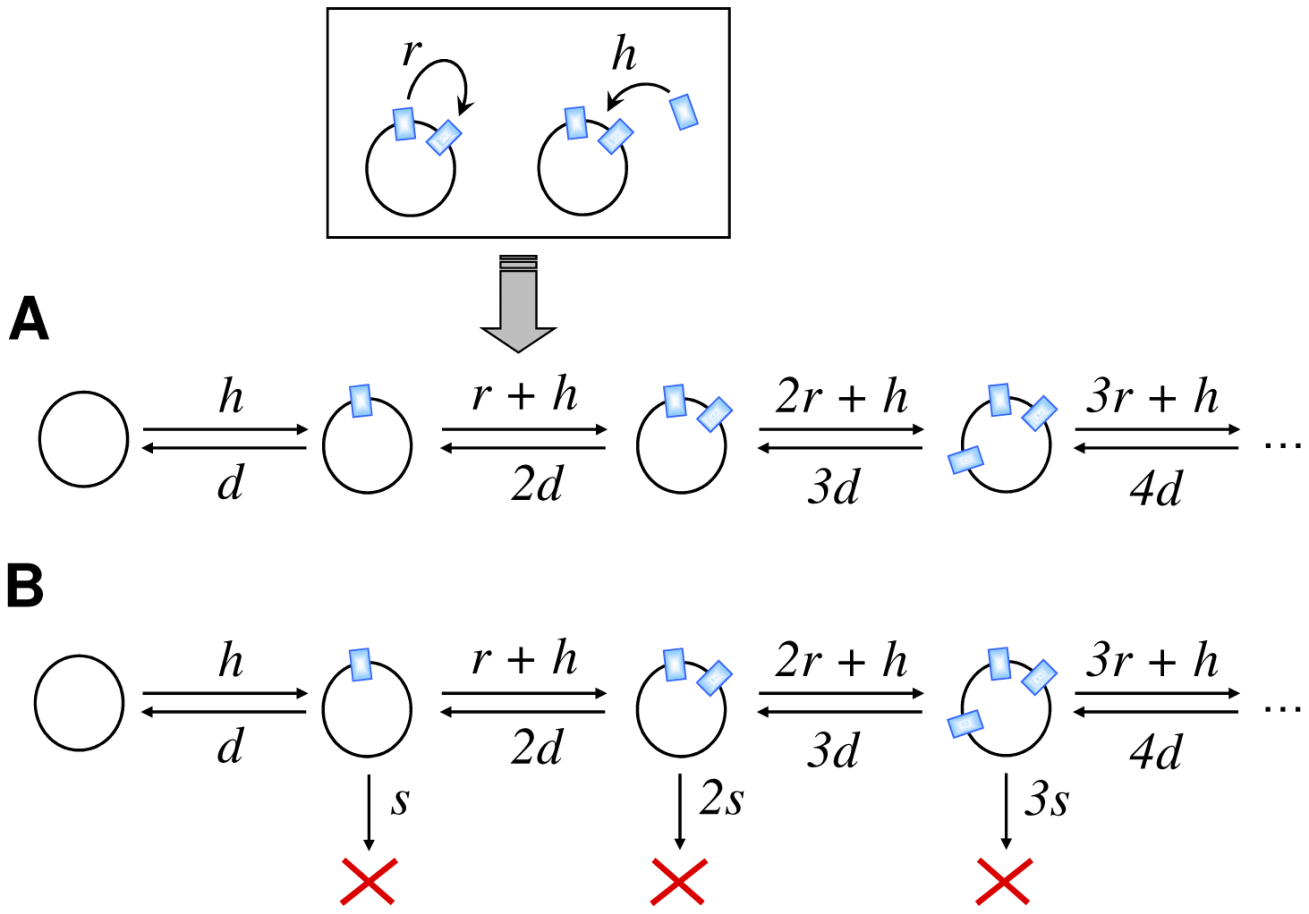
Schematic of the neutral and selection models. A. A genome containing *k* copies of an IS may increase its copy number through duplication of the extant elements, at a rate *kr*, or through lateral gene transfer, at a rate *h*. Copies are lost through deletion at a rate *kd*. B. In addition, if the IS has a fitness cost *s*, genomes subject to selection will die at a rate *ks*. Model parameters are defined as 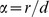 (duplicacion-deletion ratio), 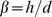 (LGT-deletion ratio), and 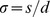 (cost-deletion ratio).

A schematic of the models is shown in Fig. 1. The rules of the models and the associated parameters should be understood in an effective manner, and in agreement with the procedure used to detect and classify IS sequences (see Methods). The duplication rate in our model applies to those insertion events that are not lethal for the host genome. From the perspective of a neutral scenario any observable insertion is assumed to be neutral or quasi-neutral: genomes hit by a lethal or highly deleterious insertion die shortly afterwards and do not further contribute to the population dynamics. The duplication parameter *r* is an effective measure of the duplication rate of an IS family. In this sense, it includes functional IS copies but also tolerates a fraction of non-functional (in the sense of non-duplicating) IS copies that might be detected in the genome and ascribed to that family. Similarly, the effective deletion parameter *d* embraces actual deletions, but also excisions that do not reinsert and sequences that, due to mutation accumulation, can no longer be detected. Finally, the LGT parameter *h* can only take into account those transfer events that conclude with the insertion of the IS in the genome. Though preventing lethal insertions of IS elements originated by duplication or LGT is a form of purifying selection, this mechanism acts on each element independently, and is thus included in the neutral model. Purifying selection that acts to streamline genomes represents a different mode of action which is included in the model incorporating selection, together with any other selective mechanism that penalizes the genome proportionally to its IS content.

The key processes in the neutral model can be summarized into two parameters: the duplication-deletion ratio *α*, and the LGT-deletion ratio *β*. The model with selection includes an extra parameter, the fitness cost-deletion ratio *σ*. The advantage of working with relative ratios becomes clear given the difficulty of obtaining reliable estimates of the actual duplication, deletion and LGT rates, which greatly vary depending on the experimental methodology and environmental conditions [8], [29], [30]. Furthermore, the duplication-deletion ratio can be easily interpreted in terms of a proliferation or deletion bias at the level of IS dynamics, as later discussed.

Both models can be solved to obtain the expected abundance distribution of an IS family in the long-term stationary state (see Methods). The models provide, for each IS family, the probability of finding a genome with a given number of copies. By comparing that probability with the observed IS abundances it is possible to estimate values for the model parameters and test whether the neutral model or the model with selection are valid to explain the genomic abundances of IS.

### Neutral evolution explains abundance and distribution of IS

Data on the classification and distribution of bacterial IS elements was taken from [31] (see Methods for further details). Starting from a dataset of 1811 bacterial chromosomes harboring at least one IS element, we selected 1079 of them by choosing randomly only one chromosome per species, in order to minimize redundancy. For each IS family, its abundance distribution was fitted to both models by means of a maximum likelihood approach.

**Figure 2:**
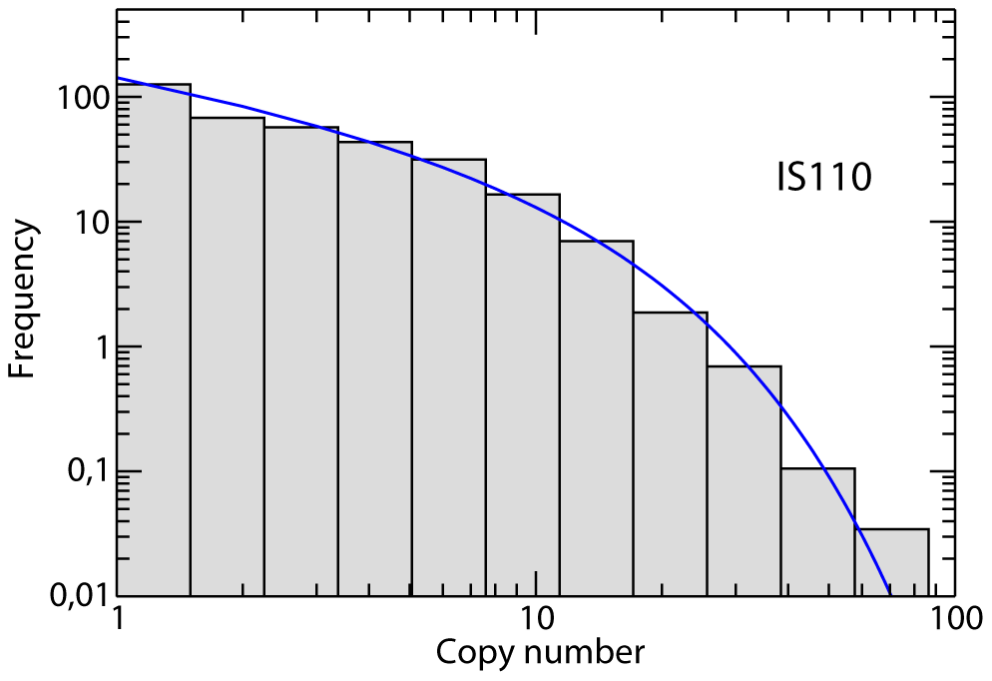
Model fit to the IS110 abundance distribution. The histogram is the empirical distribution obtained from the genomic data; the blue line is the fit to the neutral model. For this IS family, the model with selection provides a fit as good as that of the neutral model. The estimated parameters are 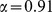, 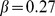 (goodness of fit 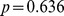).

Most of the 33 IS families show abundance distributions that are well fit by the neutral model (Fig. 2 shows a representative example). This assertion is supported by the goodness of fit tests, that render non-significant 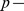
values even if no correction for multiple comparisons is applied. The only exception is IS21 (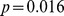), but the fit to this case becomes non-significant once corrected for the 33 comparisons. The detailed results of the fits are provided in the SI. It is remarkable that a simple, neutral model is able to explain the data with only two free parameters. We have checked whether the use of two different LGT rates, one for genomes where the corresponding IS family is absent, and a different one for genomes where the family is present improves the fits to the data. That is not the case for 31 of the 33 families, once corrected for multiple comparisons, thus suggesting that LGT rates to genomes where a given IS family is either absent or present are similar.

Next, we took the values of the duplication-deletion ratio *α* estimated in the neutral model and tried to refine the fits by adding fitness cost and selection. We found that the optimal values of the selection parameter *σ* were close to zero. In concordance, selection does not significantly improve the fit for any of the IS families (detailed results in the SI). This fact remains true even if small changes in *α* are considered. As an alternative, we also explored the selection model by adopting a completely different range of values of *α*, between 10^2^ and 10^3^, as suggested by [28]. In that scenario, duplications are overwhelmingly more frequent than deletions, and negative selection is the only factor able to prevent an explosive proliferation of the IS. As in the previous case, no improvement in the fits with respect to the neutral model is observed. It is worth mentioning that the estimated selection parameter *σ* is typically tenfold smaller than the duplication-deletion ratio.

Taken together, our results show that selection needs not be invoked to explain the abundance and distribution of IS. In the following paragraphs, we face the estimates of the neutral model to the genomic data in order to further explore the possibility that IS behave neutrally.

### Relevance of duplications and lateral transfer for IS spreading

**Figure 3:**
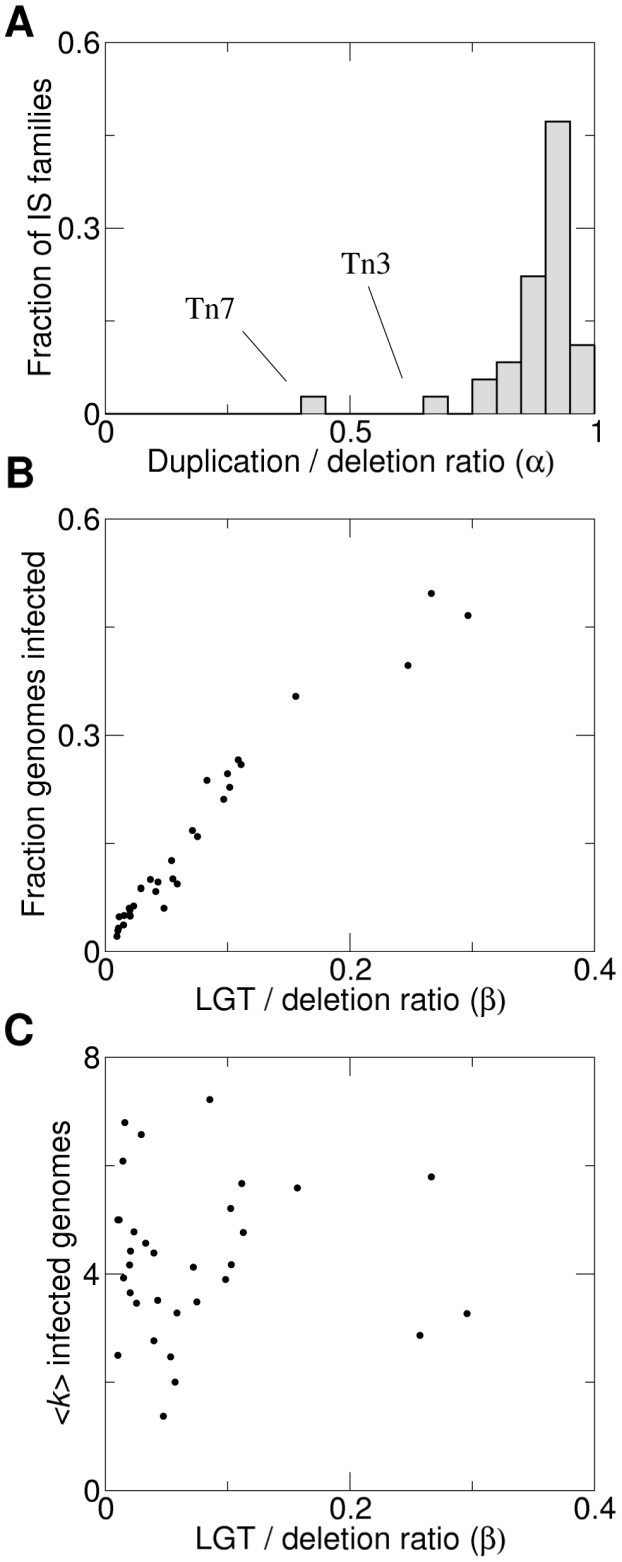
Duplication and LGT play distinct roles in IS dynamics. A. Histogram of the estimated duplication-deletion ratios (*α*) for the whole set of IS families. B. Correlation between LGT-deletion ratios (*β*) and the fraction of genomes that contain the IS family (Spearman's 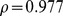, 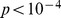, each point corresponds to an IS family). C. Lack of correlation between LGT-deletion ratios and the mean copy number within genomes with at least one copy (Spearman's 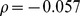, 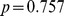).

A global analysis of the estimated parameters for the whole set of IS families reveals that most families behave in a strikingly similar way, with the duplication-deletion ratio close to 0.9 (Fig. 3(a)). Noticeable exceptions are Tn3 and Tn7, for which significantly smaller values are found.

In order to evaluate the relevance of LGT in determining the IS abundance, we studied the correlation between the LGT rates of a family (measured as parameter *β*) and the corresponding fraction of genomes that host that family (Fig. 3(b)). A strong correlation exists (Spearman's 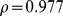, 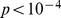), confirming the fact that the entry of new IS families into the genome totally relies on LGT. In contrast, as shown in Fig. 3(c), LGT rates do not correlate (Spearman's 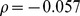, 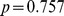) with the mean number of copies within “infected” genomes (those genomes with at least one copy for a given family). This is in agreement with the idea that duplication-deletion processes, rather than LGT, is what determines the copy number once the genome has become “infected” [26].

**Figure 4:**
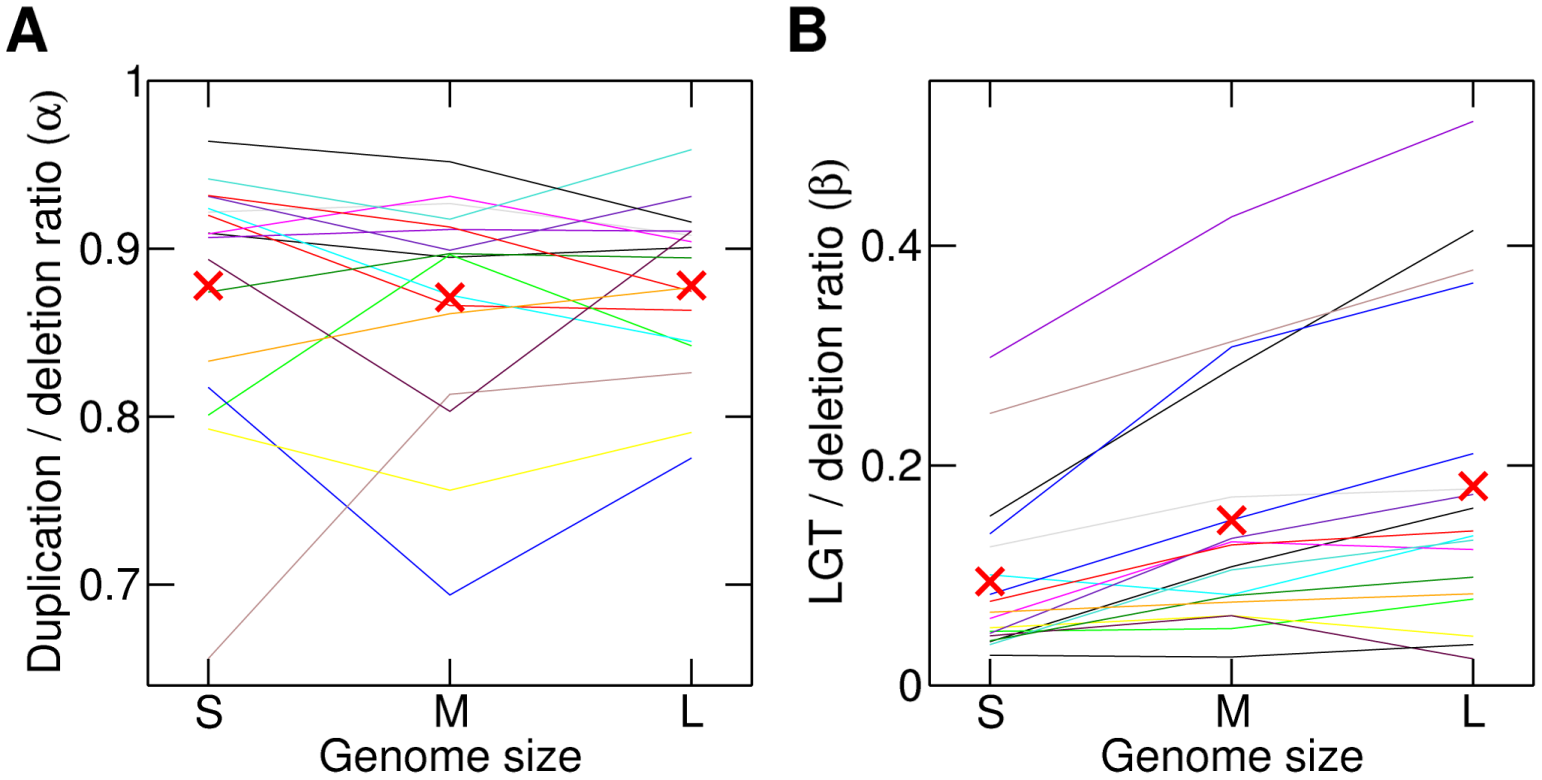
Duplication and LGT rates behave distinctly in genomes with different size. A. The duplication-deletion rate does not show a significant dependence on the genome size (Friedman test, 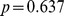). B. The LGT-deletion ratio is greater in larger genomes (Friedman test, 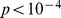). Each line corresponds to an IS family, whose parameters *α* and *β* have been estimated in small (S, <2.6 Mbp), medium (M, between 2.6 and 4.2 Mbp) and large (L, >4.2 Mbp) genomes. Red X symbols represent averages for each genome size.

We also studied whether the host genome size affects IS duplication and LGT rates. To that end, chromosomes in the database were classified into three subsets according to their sizes (smaller than 2.6 Mbp, between 2.6 and 4.2 Mbp, and larger than 4.2 Mbp). These cut-off points yield equal size subsets with approximately 350 chromosomes each. The model parameters were recalculated for each data subset and IS family (Fig. 4). We found no significant differences in the duplication-deletion ratios among the three size groups (Friedman test, 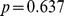). By contrast, LGT-deletion ratios show a significant increase in larger genomes (Friedman test, 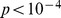). In order to complete our analysis, we also fitted the data to the selection model with a strong proliferation bias (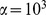) and found that the selection coefficients do not vary with the genome size (Friedman test, 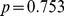).

### IS are in equilibrium inside most host genomes

A major issue concerning transposable elements is whether they can coexist with their host for long periods of time or their proliferation ultimately leads to host invasion and death. Long-term coexistence of IS and hosts becomes possible if proliferative and reductive forces compensate each other, so that the IS copy number remains stable on average. Stability is meant in a statistical sense, since the process is affected by large fluctuations. In the framework of the neutral model, this equilibrium condition can be translated into a mathematical relationship among model parameters: 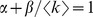, where 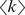 is the mean copy number of IS in the population of genomes (see SI). That expression represents a critical balance between duplication and LGT rates on the one side and deletion on the other side that permits a stable, long-lasting coexistence between IS and host (recall that 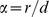 and 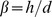). In contrast, situations where the relation above is not fulfiled lead to IS expansions or declines. Specifically, if 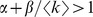, the IS proliferates “explosively”, whereas if 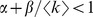, the IS gets quickly extinct.

**Figure 5:**
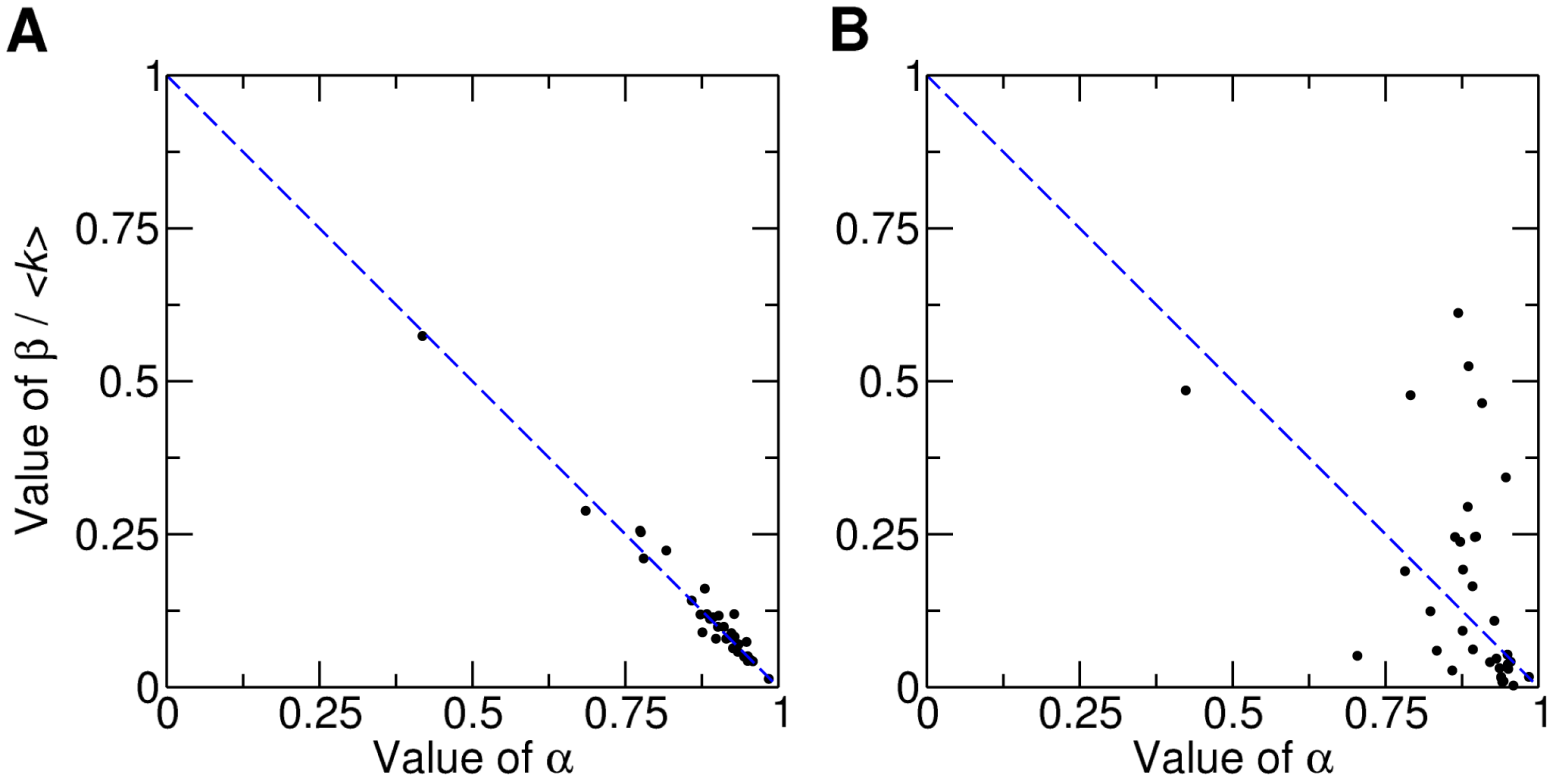
Proliferative and reductive forces are balanced in IS dynamics. Long-term coexistence of IS and hosts is possible if duplication, LGT and deletion balance according to the critical condition 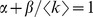 (dashed line). A. Genomic data obey the critical condition (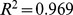). B. Simulated data resembling IS expansions do not follow the critical condition (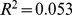).

We explored the empirical relation between the estimated parameters *α* and 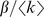 for all the IS families in the dataset. As Fig. 5(a) reveals, there is a trend of the data to be located close to the dashed line that represents the critical balance condition (coefficient of determination 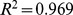). Empirical data obeying it suggest that IS and hosts have evolved stabilizing mechanisms that prevent both IS extinction and unbound proliferation in most genomes. Parameters *α* and 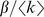 were estimated independently in order to ensure that the observed trend is not a product of the fitting algorithm (see Methods). If parameters are estimated jointly, the agreement between the empirical data and the critical balance condition rises even higher (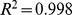).

Interestingly, this approach based on the critical balance allows for discrimination between equilibrium and IS states of exponentially fast proliferation or decline. To check for that, we generated datasets by mimicking situations where the LGT rate remains stable while the duplication rate increases (IS unbound growth) or decreases (IS decline). We found strong deviations from the critical balance, even if the simulated values of *α* and *β* were kept inside the previously observed range (Fig. 5(b)).

### Recent IS expansions are detected as outliers

The models developed in this work account for the dynamics of IS in an equilibrium state. The fact that real abundance distributions are well fit by the theoretical curves means that IS are in equilibrium in most genomes. Conversely, we can take advantage of the theoretical distributions to detect outliers, i.e. genomes that show an abnormally large copy number for a given IS family (see Methods for further details on the detection procedure). From the perspective of the neutral model, outliers can be interpreted as the result of transient imbalances in duplication, deletion and/or LGT rates, which break down the critical balance.

The search for outliers gave as a result a set of 35 strains (of a total of 1685), that span over a small number of species. It is relatively common that the same genome behaves as an outlier with respect to more than one IS family. For instance, all 12 strains of *Yersinia pestis* are outliers with respect to IS200, and three of them also with respect to IS21. Genomes belonging to the genus *Shigella* (*S. boydii*, *S. dysenteriae*, *S. flexneri* and *S. sonnei*) are overcrowded with IS1, IS3 and IS4a. Other examples are *Xanthomonas oryzae* (outlier for IS1595, IS5a, IS5b and IS701) and *Salmonella enterica* subsp. *enterica* (outlier for IS200). A summarized list can be found in Table 1, while a comprehensive list is provided in the SI.

**Table 1:** List of outlier genomes.

Species	IS families
*Acinetobacter baumanii*	IS5c, IS982
*Bordetella pertussis*	IS481
*Clavibacter michiganensis* subsp. *sepedonicus*	IS481
*Microcystis aeruginosa*	IS630
*Mycobacterium ulcerans*	IS256, ISAs1
*Salmonella enterica* serovar Typhi	IS200
*Shigella boydii*	IS1, IS3
*Shigella dysenteriae*	IS1
*Shigella flexneri*	IS1, IS3
*Shigella sonnei*	IS1, IS3, IS4a
*Streptococcus suis*	IS200
*Xanthomonas oryzae*	IS1595, IS5a, IS5b, IS701
*Yersinia pestis*	IS200, IS21

Species with an abnormally high copy number for any IS family, which reveals recent IS expansions. Typically, multiple strains per species appear as outliers (see SI for a complete list).

## Discussion

Sequencing techniques have experienced a revolution in recent years, providing researchers with an ever growing amount of data on fully-sequenced prokaryotic genomes. Nowadays, it is becoming possible to exploit all that information in order to address fundamental questions on genome evolution. In this work, we combined bioinformatics, statistical analysis and mathematical modelling of genome dynamics in order to improve our understanding of the processes that govern the spreading and extinction of transposable elements within genomes. Specifically, we focused on studying the abundance distribution of IS in bacterial genomes, and found that it can be explained as the result of a random process involving duplications, deletions and lateral transfer. Remarkably, only two parameters—the duplication-deletion ratio and the LGT-deletion ratio—are required to recover the observed distributions of all the 33 IS families considered. The simplicity of this result is surprising, considering that transposable elements are possibly engaged in a broad repertoire of intragenomic ecological-like interactions that include, among others, competition and complementation [32]–[34]. Our analysis suggests, though, that such complex interactions do not play a leading role in determining the long-term dynamics of IS in bacteria.

### Disentangling the roles of LGT, duplication and deletion

By fitting the genomic data to a neutral duplication-deletion-LGT model, we were able to observe two general trends: first, the estimated duplication rates are typically one order of magnitude greater than the estimated LGT rates; second, the LGT rate correlates with the number of genomes that host a given IS family, but does not correlate with the IS genomic abundance. These findings together let us conclude, in agreement with [26], that transposition and LGT play different roles in the dynamics of IS. Whereas LGT determines the spreading of IS across genomes, it only plays a minor role once a genome already contains a given IS family. Inside such infected genomes, the abundance of IS copies is mainly driven by stochastic duplications and deletions. When looking at the duplication-deletion ratio, we found that it takes a value slightly smaller than one, which can be interpreted in terms of a deletion bias at the level of IS [35], [36]. Such a deletion bias makes LGT essential for the long term persistence of IS: in the absence of an external income via LGT, IS copies tend to be deleted faster than they duplicate and, eventually, they disappear. This mechanism offers a possible explanation to the loss of IS in organisms whose life conditions limit their LGT rates, e.g. in anciently host-restricted endosymbionts [13].

Some authors have reported a correlation between genome size and IS content [1], [26], which motivated us to test whether duplication and LGT rates vary in genomes of different sizes. In disagreement with the prevailing idea that larger genomes withstand greater IS proliferation rates, we found no significant differences in duplication rates among genomes of different sizes. On the other hand, the LGT rate becomes greater in larger genomes (Fig. 4(b)), which opens a new path to explain the above-mentioned correlation. Actually, an observed correlation between bacterial ecology and genome size [37] suggests that prokaryotic ecological niches might be the proximate cause that determines LGT rate values.

### The role of selection

Our results show that purifying selection at the host level needs not be invoked to explain the abundance and distribution of IS, because the genomic data are fully compatible with a neutral scenario. In fact, the small differences in the distributions derived from neutral and selection models may be insufficient to discriminate between both scenarios. There are, however, some clues that challenge the prevailing role traditionally ascribed to selection. First, provided that there is a deletion bias, purifying selection is no longer essential to control IS. Second, the fact that IS in larger genomes—those with a presumably smaller fraction of essential genes—do not show reduced fitness cost challenges the view that interruption of essential genes by IS insertions generates an efficient selection pressure against IS. Third, even if there were no deletion bias and duplications greatly overwhelmed deletions, the values we found for the selection-deletion ratio—typically ten-fold smaller than the duplication-deletion ratio—bring along the possibility that IS control takes place in a weak selection scenario. This same idea had been pointed out in [28], where the abundance distribution of IS5 under the assumption of a strong proliferation bias was studied.

In a context of weak selection, the composition of the host population experiences random variations that allow for fixation of slightly deleterious genotypes [38]. Hence, when the host population dynamics is taken into account, opposite predictions are derived from deletion and proliferation biased scenarios (see Table 2). In the former case, the IS copy number is controlled by deletions, and selection may be neglected, thus resulting in an effectively neutral dynamics. In the latter case, explosive IS proliferation would be the expected outcome because weak purifying selection is unable to compensate for IS duplications (see the SI for analytical calculations). Therefore, finding weak selection rates in a proliferation biased scenario necessarily implies that host genomes are out of equilibrium and in their way to becoming fully invaded by IS [12], [25].

**Table 2:** Expected behavior of the IS abundance under alternative proliferation dynamics and evolutionary conditions.

	Evolutionary condition		
IS dynamics	Stable conditions	No LGT	Changing environment
Neutral, deletion bias	Active IS in equilibrium. Abundant degenerated copies	Loss of active IS, vestigial copies can remain	Instability in IS copy number if duplication, deletion and LGT rates are imbalanced
Weak selection, proliferation bias	IS explosion	IS explosion	IS explosion
Efficient selection, proliferation bias	Active IS under control, few or no non-functional copies	Loss of active IS, few or no non-functional copies	IS explosion if population size decreases

Alternative hypotheses about the factors that govern IS dynamics yield distinct predictions on the abundance of IS under different evolutionary conditions. The case of deletion bias with selection has not been considered, because in such a case the role of selection in controlling IS becomes secondary.

### A neutral, punctuated scenario for IS dynamics

At odds with the aforementioned scenario of non-equilibrium proliferative dynamics, our results point towards a stable coexistence of IS and hosts. Despite the fact that molecular mechanisms of transposition vary [9], all of the 33 IS families considered show strikingly similar values of the dynamical parameters. Even more, duplication, deletion, and LGT rates balance according to a critical condition that allows for evolutionary persistence without explosive proliferation. Such a narrow range of parameter values suggests an implicit role of stabilizing selection acting on IS and promoting those that behave like mild, persistent parasites [39]. In fact, IS mutants that fall below the critical condition are doomed to disappear; those that excede it proliferate quickly and—even if they entail a minimal fitness cost—eventually kill their local host populations, thus causing their own extinction [40].

Degenerated IS copies constitute a hallmark of the neutral dynamics based on deletion bias: IS are controlled via deletions, which turn functional IS copies into degenerated (or vestigial) ones. In contrast, if IS are to be controlled via purifying selection, whole genomes rich in IS tend to disappear, without generation of any IS remnants. On this point, it is worth discussing the case of *Wolbachia*, a genus of anciently host-restricted endosymbiotic bacteria. *Wolbachia* endosymbionts have reduced genomes (∼1 Mbp) and their effective population sizes are thought to be very small. The strains of *Wolbachia* that are associated to arthropods (e.g. *Drosophila melanogaster* and *Culex quinquefasciatus*) are known to coinfect hosts and undergo LGT [41], [42]; while those associated to filarial nematodes (*Brugia malayi* and *Onchocerca ochengi*) seem to be transmitted in a strictly vertical way, which greatly limits LGT [43]. In agreement with the idea that LGT is essential for the maintenance of IS, only the arthropod-associated *Wolbachia* strains host functional IS copies [44], [45]. Importantly, the comparative analysis of IS in *Wolbachia* reveals that more than 70% of IS copies in arthropod-associated strains are nonfunctional [27], [46]. Those nonfunctional copies belong to several IS families, which are also represented in nematode-associated *Wolbachia* with no functional copies. Large amounts of partial IS copies have also been found in a recent study dealing with thermophilic cyanobacteria of the genus *Synechococcus* [47]. These facts suggest that nonfunctional, fragmentary IS copies may be prevalent in bacterial genomes, even if they have experienced strong reductions in size, and that deletions are an important force leading to the loss of IS. In contrast, group II introns—another kind of TE in prokaryotes—display a smaller fraction of fragmentary copies and their dynamics are possibly driven by selection [48].

The neutral dynamics that we present here can give rise to punctuated events of IS proliferation. They occur whenever the LGT, duplication and deletion rates become imbalanced and the critical condition breaks down. We have identified some of those events by applying an outlier detection algorithm on the abundance distributions. According to our analysis, the fraction of such outliers is small, hence confirming that non-equilibrium states are the exception rather than the rule. Some of the outliers that we have detected have already been noticed and interpreted in the literature as IS expansions [14], supporting the idea that outliers truly represent genomes that have experienced an episode of IS proliferation. It is not rare that multiple IS families show expansions within the same genome, which suggests that the causes of IS punctuations do not lie at the IS but at the host level. Indeed, some IS expansions have been associated to episodes where bacteria underwent host restriction [11], [13], [14]. Traditionally, the reduced efficiency of purifying selection in smaller populations has been invoked to explain such expansion events. There are other mechanisms, though, that may account for IS punctuations in the absence of selection. Transitory alterations in the deletion and LGT rates may play the same role, as well as stress induced downregulation of host regulatory mechanisms limiting IS transposition [17], [29]. In an indirect way, ecological changes—such as host restriction—may imply reductions in the fraction of essential genes [49], [50], which would lead to a higher probability of IS insertions being non-lethal, and eventually to increases in the effective duplication rate [26].

### Conclusions

In sum, our results indicate that the persistence of IS in bacterial genomes are the outcome of a neutral process, with little role for purifying selection. Let us emphasize that the absence of selection here reported should be interpreted as a general trend in the whole set of genomes, averaged over long periods of time. Sporadic cases of IS insertions affected by selection may occur, but the neutral behavior dominates at large evolutionary scales. Most genomes contain IS abundances compatible with an equilibrium state, albeit punctual imbalances in the LGT, duplication and deletion rates—but not necessarily in the host population size—may produce transient IS expansions. In the light of the important role of transposable elements in adaptation and genome evolution [4], [6], [17], [51], a better understanding of the actual causes behind IS expansions becomes an appealing challenge. From an “ecological” perspective, most IS families share closely similar values of the relevant dynamical parameters, suggesting that IS and host genomes have coevolved towards a state of stable coexistence. The apparent equivalence of different IS families brings to mind the concept of a neutral ecosystem [52]. Hence, it would be interesting to further explore the parallelisms between IS dynamics and neutral ecology, which could provide us with novel insights into the processes that rule the architecture of genomes.

## Methods

### IS dataset: Detection and classification methodology

We used the catalog in [31] as a source of information regarding bacterial IS classification and distribution. IS catalog construction is briefly summarized in the following. In a preliminary study, transposases and other IS-encoded proteins collected from Pfam (v2.6) [53] and ISfinder [54] (a specialized database focused on prokaryotic IS elements) were used to generate a manually curated list of protein architectures (protein domain organization descriptions) associated to IS-encoded proteins. Listed architectures represented, by extension, IS-associated genes. Simultaneously, a table describing the correspondence between gene combinations (represented by protein architecture strings) and IS elements classified according the the IS finder classification scheme, was built. Then, chromosomal and predicted protein sequences, as well as protein translation tables (gene coordinate files) for 2074 bacterial chromosomes were downloaded from the NCBI Genome database on October 2012.

A computational pipeline written in Perl directed the execution of HMMER 3.0 and other in-house developed applications to detect, classify and count IS elements in complete genomes. First, the protein architecture for the complete set of proteins predicted for all bacterial genomes was reconstructed on the basis of HMMER alignments against the Pfam database. Then, IS-associated genes were identified by comparison with the previously generated list of protein architectures. Once IS-associated genes had been identified, the system assigned individual genes, or clusters of adjacent genes, to IS elements using the correspondence table also established in the preliminary study.

The system attempted to resolve IS elements located in tandem, as well as to identify complete IS elements that could exist within gene clusters originated by nested insertions. To do so, clusters of IS-associated genes were segmented into all possible collections of adjacent gene subclusters, which were then classified, when possible, as belonging to a certain IS family. The segmentation scheme used maximized the total length of successfully classified subclusters. As result, 69,438 IS associated genes, corresponding to 57,515 IS elements in 1,1811 chromosomes, were identified. The overall IS detection and classification strategy aimed at reducing the number of wrongly classified genes at the expense of a slight decrease in sensitivity. With this purpose, the system was based on NCBI published gene predictions and only individual or adjacent gene clusters that could be unequivocally assigned to IS elements belonging to canonical IS families or groups were considered.

Two approaches were followed to evaluate the quality of the annotations generated by the IS detection and classification pipeline. For the first approach, the set of genes annotated in the NCBI database as encoding for transposases was compared against the set of IS-associated genes detected by the pipeline. Out of the 65,230 genes annotated with the keyword ‘transposase’ at the NCBI database, 85% were correctly identified by the pipeline. For the second approach, IS family affiliation was compared for the sets of IS-associated genes described both in the genomic component of ISfinder (ISbrowser [55]) and in the annotations generated by the pipeline. At a global level, IS family affiliation agreed for 88% of the 866 shared IS-associated genes. At the level of individual IS families, the fraction of genes that were affiliated to the same IS family by both systems had average and median values of 79% and 100%, respectively.

### Neutral model

We studied the neutral evolution of the number of copies in the genome as a generalized birth and death process (Fig. 1(a)). A complete analysis of this kind of processes applied to the study of proteomes has been carried out in [56].

The neutral model focuses on a particular IS family in a single genome. Elements belonging to the family are duplicated at a rate *r* and deleted at a rate 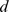. In addition, new copies can be inserted through lateral transfer at a rate 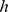. We define the state of the genome as the number of copies that it carries, with no upper limit for such copy number.

A genome with 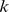 copies will turn into a state with 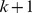 copies after duplication or LGT. Under the assumption that copies behave independently and LGT rate is a constant, the transition rate 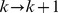 is equal to 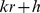. On the other side, the transition rate 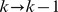 due to copy deletion is equal to 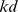. As described in Fig. 1, the relevant parameters in this case are 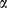 (duplication-deletion ratio) and 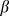 (LGT-deletion ratio). From a formal perspective, working with those ratios simply amounts to measuring time in units of IS deletion events.

The duplication, deletion and transfer processes reach a stationary state where the probability 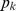 of finding a genome with 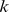 copies follows [56]

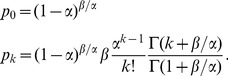
(1)


The duplication-deletion ratio, 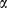, plays a central role in the dynamics. If 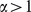 the number of copies inside the genome increases steadily until it invades the genome. This proliferation-biased scenario, is unrealistic in the absence of purifying selection. In contrast, if 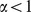 duplications are slower than deletions and the copies inside the genome tend to disappear. In this deletion-biased scenario the extinction of the IS is prevented by the external contribution of lateral transfer.

### Model with selection

Adding selection to the model requires considering a whole population of genomes instead of a single genome. Inside each genome the dynamics of duplication, deletion and lateral transfer remains the same as in the neutral model. In addition, the IS copy number 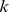 determines a fitness cost 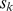 on the host genome. A schematic of the resulting process is depicted in Fig. 1(b). For simplicity we assume that the fitness cost is linear in the number of copies, 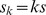, and define the cost-deletion ratio 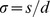. From a mathematical point of view, the model with selection can be seen as a multitype branching process whose stationary behavior is described by its generating matrix 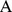 [21], [28].
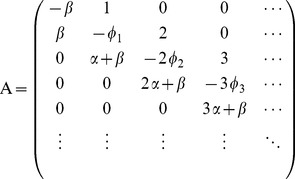
(2)where 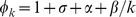.

The population evolves according to the following dynamical equation:
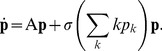
(3)


The stationary composition of the population is described by the eigenvector 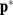 associated with the greatest real eigenvalue of 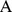. The stationary abundance distribution 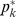 is equal to the 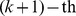 component of 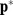. (Note that 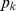 takes values from 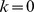, which corresponds to the first component of 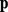). It is worth mentioning that the neutral model can be derived from the selection model in the limit 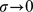.

### Data pre-processing

In order to compare the genomic data with the models we assume that the dynamics of a particular IS family is similar in all genomes, while different families behave independently. Therefore, the genomic frequencies observed for a given IS family can be interpreted on the basis of the probability 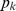 of finding a genome with 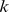 copies in a population of independent genomes. This is an assumption supported by empirical and theoretical arguments. Indeed, even in strains of the same species, the abundance of an IS family varies broadly [27]. On the theoretical side, IS dynamics are a kind of branching processes [57], where information on initial conditions is lost exponentially fast [58] –for all practical purposes, within a few duplication cycles. This nonetheless, and in order to minimize the possible bias introduced by closely related strains, we restricted our analysis to a dataset composed of only one strain per species. Although genomes from distinct species may be not completely independent, the averaging on many non-related groups compensates for that. As a confirmation, taking one genome per genus and repeating the analysis did not change our results. The full dataset with multiple strains per species was only used to detect outliers.

Absence of a particular IS family in a genome may have two causes. One is the dynamics described by our models, which include the temporal extinction of an IS family. Another one is the possibility, that we cannot discard, that a specific genome is non-invadable by that family. Since we cannot distinguish between both mechanisms, we excluded from this study those genomes which do not contain any IS family at all. The remaining dataset (provided as Table 4 in the SI) contains 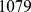 bacterial chromosomes (harboured by 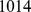 species). As it is quite a large number, special cases of genomes that may be non-invadable by certain IS families are not expected to introduce a significant bias into the estimation of 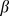. Alternatively, IS familes that are very specific to certain genomes can be detected through their poor fits.

### Parameter estimation, goodness of fit test and model comparison

IS families that appear in fewer than 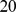 genomes were discarded to prevent unreliable estimates associated to small datasets. The following parameter estimation was done independently for each of the 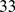 remaining IS families. First, the frequency distribution of the family was extracted from the genomic data. Then, for each model a maximum likelihood approach was applied to determine the parameters that best fit the model to the data. As a numerical optimization algorithm, we used the simplex method implemented in *MATLAB* (MATLAB version 7.6.0.324 (R2008a). Natick, Massachusetts: The Mathworks Inc.). The robustness of our qualitative results against the split of IS families into different subfamilies was also tested. Additional fits to IS copy number abundance were carried out for three families that can be clearly separated into groups: IS4, IS5 and IS66.

Some care must be taken in order to evaluate the role of selection. The key difficulty is the fact that parameter estimation in the selection model is confused by multiple local maxima in the likelihood function. Since local maxima with similar values are distributed along the whole parameter range, parameter estimation becomes strongly dependent on their initial guesses. As a result, an *a priori* estimation of some parameters is required before the selection model can be fitted to the data. Because the neutral model is a particular case of the selection model, we took 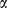 from the neutral setting and tried to refine the fit by adding selection. Alternatively, we explored the selection model by choosing a qualitatively different range of values of 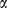, between 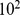 and 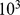 (as suggested in [28]); and also the case of a small (but greater than one) 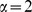.

The goodness of the fits was evaluated by means of a likelihood ratio test that compared the observed and expected frequencies for each abundance interval. This test is similar to a Chi-square test, but more suitable if any of the differences between the observed and expected frequencies is greater than the expected frequency. Different abundance intervals have been defined for each IS family in such a way that at least two occurrences are expected for each interval (alternative criteria have been tried without major changes in results). The 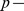
values associated to the test statistics have been numerically computed by simulating a sampling process on the expected distribution. Comparison between neutral and selection models was done in terms of the corrected Akaike Information Criterion [59], both models containing two degrees of freedom (because 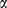 is fixed in the model with selection).

The detailed results of the fits to the neutral and selection models are provided in the Supplementary Information.

### Detection of outlier genomes

For each IS family, outliers are genomes that contain a large copy number, so large that it cannot be explained by any of the models. Specifically, let us define 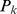 as the probability of having 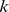 or more copies, 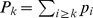. The probability that a genome with 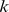 or more copies is found in a sample of 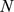 genomes is 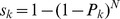. The value of 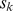 is indeed the significance level, already corrected by the sample size [60]. It can be set to the desired value in order to numerically obtain the copy threshold 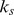. Thus, genomes with more than 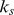 copies are outliers at a corrected significance level 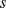. Copy thresholds are different accross IS families, thus detection of outliers was carried out independently for each family. We tried 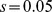 and 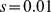 with similar results. As we looked for outliers in the full dataset (including more than one strain per species), we took a sample size 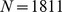 chromosomes. That is a conservative choice, since the actual number of independent instances in the dataset may be smaller; however, similar results were obtained by setting 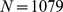 (the number of different species). Notice that the correction for sample size implies that the significance threshold per genome, in all these conditions, is close to 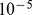.

### Independent estimation of *α* and *β*

The critical condition 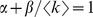 sets an implicit constraint if a stationary abundance distribution is to be established. When it comes to study the condition above, such a constraint may give rise to a false correlation if the fitting algorithm estimates 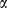 and 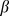 jointly. In order to avoid the introduction of spurious correlations, we used an alternative approach that provides an independent (although less precise) estimation of the parameters. First, the LGT-deletion ratio was estimated as 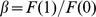, where 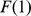 and 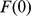 are the frequencies of genomes with one and no copies, respectively. Next, we discarded genomes with no copies and estimated *α* only from “infected” genomes. These parameter values were used to test the critical balance. By simulating non-stationary genomes we checked that the independent estimation algorithm does not give rise to false correlations.

## Supporting Information

Dataset S1 Excel file with a list of genomes analysed and corresponding abundances of all IS families. A black mark left of the genome name indicates those genomes included in the non-redundant dataset.
(XLS)

Text S1 This file contains the following information: (i) summary of fits to data (section S1) and details for the neutral model (Table S1), the selection model (Table S2), and the fits to complete families IS4, IS5 and IS66 (Table S3); (ii) derivation of the critical condition (section S2); (iii) comprehensive list of outlier genomes (Section S3 and Table S4); (iv) a discussion on the effect of weak selection on the IS copy number (section S4).
(PDF)
