# Supplementary material for: Large-Scale Genomic Analysis Suggests a Neutral Punctuated Dynamics of Transposable Elements in Bacterial Genomes
Source: PLoS Comput Biol. 2014 Jun 26;10(6):e1003680. doi: 10.1371/journal.pcbi.1003680 (PMC4072520; doi:10.1371/journal.pcbi.1003680)
Supplement: Text S1 — This file contains the following information: (i) summary of fits to data (section S1) and details for the neutral model (Table S1), the selection model (Table S2), and the fits to complete families IS4, IS5 and IS66 (Table S3); (ii) derivation of the critical condition (section S2); (iii) comprehensive list of outlier genomes (Section S3 and Table S4); (iv) a discussion on the effect of weak selection on the IS copy number (section S4). (PDF) [file pcbi.1003680.s002.pdf]

SUPPLEMENTARY INFORMATION – LARGE-SCALE GENOMIC ANALYSIS SUGGESTS A NEUTRAL PUNCTUATED DYNAMICS OF TRANSPOSABLE ELEMENTS IN BACTERIAL GENOMES

JAIME IRANZO, MANUEL J. GÓMEZ, FRANCISCO J. LÓPEZ DE SARO AND SUSANNA MANRUBIA

S1. Results of the fits to the neutral and selection models

The results of the fits to the neutral model are shown in Table S1. It shows the fit parameters and the p-value associated to the goodness of fit test for two variants of the model: either with a single LGT rate β ("One LGT rate", as explained in the main text) or with different LGT rates in empty and IS hosting genomes ("Two LGT rates", parameter β_0 accounts for LGT rate in empty genomes). The latter scenario would account, for instance, for IS families that provide the host genome with some kind of resistance against the entry of additional IS copies. The last column in the table contains the difference in the corrected Akaike Information Criterion for both models (with two and three degrees of freedom, respectively). Since we are dealing with nested models, the ΔAICc is equivalent to a likelihood ratio test and follows a Chi-squared distribution with one degree of freedom. Therefore, ΔAICc > 6.64 implies that the "two LGT rates" model is more probable to be true at a (non-corrected by multiple comparisons) significance level of 0.01.

Table S2 contains the results of the fit to the model with selection. The case where the value of α was taken from the neutral fit rendered selection parameters $\sigma \leq 10^{-5}$ and it is not included in the table. In the scenarios with $\alpha = 10^3$ and $\alpha = 2$, the ΔAICc values show that the fits to the model with selection are as good as—but not better than—those to the neutral model.

The fits presented in Table S3 have been obtained after merging the data from subgroups of families IS4, IS5 and IS66. They demonstrate that joining the subgroups within each family does not change our results. Specifically, (a) α values are similar and β values are approximately the sum of those in subgroups; (b) the critical relation also holds; (c) σ values are similar and no qualitative changes in ΔAICc are found.

S2. Derivation of the critical condition $\alpha + \beta/\langle k\rangle = 1$

Let us consider the scenario defined by the free-parameter neutral model. The copy number distribution in such a scenario is defined by equation (1) in the main text. In correspondence, the average number of copies per genome for a particular IS family is equal to

$$\langle k\rangle = \frac{\beta\,\beta_0}{(1-\alpha)\left(\beta_0 + (\beta-\beta_0)(1-\alpha)^{\beta/\alpha}\right)} \tag{1}$$

Date: March 27, 2014.

Let us explore the possibility that the LGT rate is proportional to the abundance of IS, expressed as its average copy number. Specifically, we write $\beta = \gamma\langle k\rangle$ and $\beta_0 = \gamma_0\langle k\rangle$, where the proportionality constants γ and γ_0 will be called the relative LGT-deletion ratios. Substitution of these assumptions in eq. (1) results in a fixed point equation, that after some manipulation takes the following form:

$$(\gamma_0 - \gamma)(1-\alpha)^{\gamma\langle k\rangle/\alpha} = \gamma_0\left(1 - \frac{\gamma}{1-\alpha}\right) \tag{2}$$

If both LGT rates are equal, then $\gamma_0 = \gamma$. In such a case, a stationary state where $\langle k\rangle$ is finite and greater than zero can only be reached if

$$0 = \gamma\left(1 - \frac{\gamma}{1-\alpha}\right)$$

what leads to the condition $\alpha + \gamma = 1$.

Therefore, in the neutral model with LGT-deletion ratio proportional to the average copy number and $\beta = \beta_0$, the relation $\alpha + \gamma = 1$ determines the critical condition for the stability of the system. If $\alpha + \gamma < 1$ the IS's will become extinct, whereas if $\alpha + \gamma > 1$ an explosive proliferation of copies will take place.

S3. Outlier genomes

Table S4 contains the list of all genomes detected as outliers according to the algorithm described in the main text, Methods section. The fact that a genome behaves as an outlier implies that it hosts an abnormally high copy number for any IS family, which possibly results from recent IS expansions. The same bacterial strain can behave as an outlier with respect to more than one IS family.

S4. Transposition ratchet in small populations

In this section, the effect of weak selection on the IS copy number is analized. Even if selection acts against the expansion of IS's, the stochastic dynamics in finite populations of genomes may lead to the fixation of genomes with greater copy number.

Let us consider a Moran process on a population with effective size N. Moreover, we will assume that the duplication rate $r \ll N^{-1}$, and the same for the deletion rate d. Under this assumption, the copy number in the population is homogeneous and mutants with increased (or reduced) copy number either become fixed or get extinct before the next mutant appears.

Starting with an homogenous population of genomes that contain k copies, we are interested in obtaining the probabilities that the population evolves towards states with $k+1$ and $k-1$ copies. Let us denote those probabilities as ρ_k^+ and ρ_k^-, respectively. We focus on the case where the genome fitness decreases linearly with the copy number as $f_k = 1 - sk$ (here s is the fitness cost of a single copy). For the Moran process considered, the transition probabilities can be calculated analytically as the product of the mutation rate and the fixation probability:

$$\rho_k^+ = kr \frac{1 - f_k/f_{k+1}}{1 - (f_k/f_{k+1})^N} = \frac{kr}{N}\left(1 - \frac{N-1}{2}\, s\right) + \mathcal{O}(Ns)^2 \tag{3}$$

$$\rho_k^- = kd \frac{1 - f_k/f_{k-1}}{1 - (f_k/f_{k-1})^N} = \frac{kd}{N}\left(1 + \frac{N-1}{2}\, s\right) + \mathcal{O}(Ns)^2 \tag{4}$$

In conditions of weak selection, $s \ll N^{-1}$, the terms of higher order in Ns can be neglected. Thus, it is straightforward to obtain the ratio between both transition probabilities, which does not depend on the actual copy number:

$$\frac{\rho^+}{\rho^-} = \alpha\,(1 - (N-1)\,s) \tag{5}$$

where $\alpha = r/d$ is the duplication-deletion ratio.

An interesting consequence is the prediction of IS expansions in finite populations when selection is weak and $\alpha \gg 1$ (notice that this holds even if there is no LGT). This phenomenon may allow for discrimination between the supercritical and subcritical scenarios. In the former case (characterized by $\alpha \gg 1$) selection, even if weak, is essential for controlling the copy number. In the latter, deletion alone compensates for IS proliferation and weak selection, even if present, can be neglected without qualitative consequences.

Centro de Astrobiología, INTA-CSIC, Torrejón de Ardoz, Madrid, Spain

Table S1. **Fit of the data to the neutral models.**

		One LGT rate			Two LGT rates				
	N	α	β	p	α	β	β_0	p	ΔAICc
IS1	46	0.95	0.01	0.44	0.97	-0.18	0.02	0.48	-0.77
IS110	529	0.91	0.27	0.53	0.88	0.46	0.22	0.64	5.00
IS1182	227	0.88	0.10	0.34	0.90	0.02	0.10	0.36	-1.46
IS380	67	0.92	0.02	0.54	0.92	0.06	0.02	0.53	-2.15
IS1595	135	0.86	0.06	0.35	0.94	-0.33	0.08	0.63	**9.33**
IS1634	34	0.93	0.01	0.58	0.87	0.41	0.01	0.58	-0.48
IS200	422	0.77	0.26	0.29	0.84	0.02	0.30	0.52	5.00
IS200/IS605	105	0.78	0.05	0.58	0.72	0.35	0.05	0.58	-1.21
IS21	498	0.81	0.30	**0.02**	0.89	-0.08	0.39	0.33	**23.9**
IS256	376	0.92	0.16	0.59	0.93	0.11	0.16	0.57	-1.56
IS3	702	0.91	0.40	0.29	0.92	0.38	0.41	0.31	-1.91
IS30	243	0.89	0.10	0.46	0.91	-0.01	0.11	0.53	-0.67
IS481	63	0.87	0.03	0.51	9,86	0.09	0.02	0.51	-2.11
IS4a	57	0.91	0.02	0.68	0.91	0.05	0.02	0.67	-2.21
IS4b	40	0.90	0.02	0.50	0.82	0.42	0.01	0.52	-0.31
IS5a	264	0.92	0.10	0.53	0.94	-0.02	0.12	0.60	0.22
IS5b	285	0.93	0.11	0.61	0.90	0.33	0.09	0.48	3.83
IS5c	54	0.91	0.02	0.43	0.94	-0.18	0.02	0.57	-0.92
IS5d	53	0.95	0.02	0.60	0.96	-0.01	0.02	0.75	-2.21
IS6	103	0.87	0.04	0.54	0.86	0.12	0.04	0.45	-1.87
IS607	52	0.88	0.02	0.54	0.85	0.17	0.02	0.58	-1.86
IS630	253	0.95	0.08	0.67	0.95	0.04	0.09	0.73	-1.75
IS66a	177	0.90	0.07	0.64	0.90	0.07	0.07	0.64	-2.07
IS66b	31	0.93	0.01	0.14	0.98	-0.42	0.02	0.51	2.60
IS701	106	0.91	0.04	0.26	0.91	0.04	0.04	0.61	-2.12
IS91	86	0.82	0.04	0.43	0.92	-0.35	0.05	0.64	4.15
IS982	94	0.95	0.03	0.50	0.94	0.13	0.03	0.55	-1.56
ISAs1	91	0.92	0.03	0.48	0.88	0.26	0.03	0.54	-0.25
ISAzo13	22	0.80	0.01	0.50	0.70	0.31	0.01	0.58	-2.27
ISL3	277	0.91	0.11	0.41	0.93	-0.03	0.13	0.54	1.10
ISNCYa	166	0.86	0.08	0.23	0.64	1.00	0.05	0.54	**24.0**
Tn3	98	0.69	0.06	0.52	0.48	0.54	0.05	0.54	1.25
Tn7	64	0.42	0.05	0.55	0.31	0.22	0.05	0.56	-1.99

Parameter β_0 is the LGT rate to empty genomes. The p-values correspond to the goodness of fit tests. ΔAICc > 6.64 implies that the second model is more probable to be true at a (non-corrected) significance level of 0.01. After correction for multiple comparisons, only IS21 and ISNCYa show a significant ΔAICc.

TABLE S2. **Fit of the data to the selection models.**

	$\alpha = 10^3$			$\alpha = 2$		
	β	σ	ΔAICc	β	σ	ΔAICc
IS1	15	57	-2.6E-5	0.03	0.06	-2.9E-5
IS110	293	102	4.2E-6	0.59	0.11	3.1E-6
IS1182	112	135	1.1E-5	0.22	0.15	8.8E-6
IS1380	25	85	6.4E-6	0.05	0.09	5.3E-6
IS1595	68	164	1.9E-6	0.14	0.19	1.3E-6
IS1634	12	77	4.4E-7	0.02	0.08	-1.8E-6
IS200	333	294	8.3E-6	0.67	0.36	8.0E-6
IS200/IS605	69	283	1.8E-6	0.14	0.35	1.6E-6
IS21	367	241	5.2E-6	0.73	0.29	4.7E-6
IS256	171	87	9.8E-6	0.34	0.09	8.3E-6
IS3	436	95	1.7E-6	0.87	0.10	1.4E-6
IS30	116	122	3.2E-6	0.23	0.14	9.5E-7
IS481	29	144	1.5E-6	0.06	0.16	1.3E-6
IS4a	22	95	6.0E-6	0.04	0.10	5.3E-6
IS4b	17	114	2.7E-6	0.03	0.13	2.0E-6
IS5a	111	86	1.7E-5	0.22	0.09	1.0E-5
IS5b	120	80	4.6E-6	0.24	0.09	1.1E-6
IS5c	22	104	1.0E-6	0.04	0.11	-1.4E-6
IS5d	17	49	-1.9E-4	0.03	0.05	-2.0E-4
IS6	49	145	3.3E-7	0.10	0.16	-3.7E-7
IS607	23	130	3.2E-6	0.05	0.14	2.8E-6
IS630	90	53	-6.8E-4	0.18	0.06	-6.8E-4
IS66a	80	118	1.9E-6	0.16	0.13	-1.6E-6
IS66b	11	76	1.7E-5	0.02	0.08	1.5E-5
IS701	44	100	2.2E-5	0.09	0.11	2.0E-5
IS91	48	217	1.6E-6	0.10	0.26	1.4E-6
IS982	31	53	-1.6E-4	0.06	0.06	-1.6E-4
ISAs1	36	93	2.5E-6	0.07	0.10	2.9E-7
ISAzo10	13	252	3.4E-6	0.03	0.30	3.3E-6
ISL3	124	100	9.3E-6	0.25	0.11	6.5E-6
ISNCYa	87	160	1.5E-6	0.17	0.18	2.6E-7
Tn3	83	450	5.8E-7	0.16	0.59	5.6E-7
Tn7	113	1368	2.3E-7	0.22	2.16	2.2E-7

The ΔAICc values correspond to the comparison between the neutral model and the models with selection.

Table S3. **Additional fits for families IS4, IS5 and IS66.**

(a) Neutral model	N	α	β	p
IS4 (a+b)	97	0.903	0.037	0.653
IS5 (a+b+c+d)	506	0.934	0.221	0.771
IS66 (a+b)	195	0.908	0.077	0.642

(b) Critical relation	N	α	β	$\beta/\langle k\rangle$
IS4 (a+b)	97	0.910	0.034	0.089
IS5 (a+b+c+d)	506	0.935	0.218	0.065
IS66 (a+b)	195	0.904	0.080	0.097

(c) Selection, $\alpha = 10^3$	N	β	σ	ΔAICc
IS4 (a+b)	97	41	106	2.2E-6
IS5 (a+b+c+d)	506	237	71	-6.2E-5
IS66 (a+b)	195	84	102	3.0E-6

The ΔAICc values correspond to the comparison between the neutral model and the model with selection. In order to test the critical relation, a modified algorithm is used to obtain independent estimates of α and β.

TABLE S4. **Full list of outlier genomes.**

Strain name	IS families
Acinetobacter baumanii SDF	IS5c (138), IS982 (141)
Bordetella pertussis CS	IS481 (199)
Bordetella pertussis Tohama I	IS481 (215)
Clavibacter michiganensis subsp. *sepedonicus*	IS481 (89)
Microcystis aeruginosa NIES-843	IS630 (116)
Mycobacterium ulcerans Agy99	IS256 (71), ISAs1 (185)
Salmonella enterica serovar Typhi str. CT18	IS200 (27)
Salmonella enterica serovar Typhi str. P-stx-12	IS200 (27)
Salmonella enterica serovar Typhi str. Ty2	IS200 (27)
Shigella boydii CDC 3083-94	IS1 (194)
Shigella boydii Sb227	IS1 (171), IS3 (132)
Shigella dysenteriae Sd197	IS1 (477)
Shigella flexneri 2002017	IS1 (107), IS3 (96)
Shigella flexneri 2a str. 2457T	IS1 (111)
Shigella flexneri 2a str. 301	IS1 (116), IS3 (108)
Shigella flexneri 5 str. 8401	IS1 (110)
Shigella sonnei 53G	IS1 (172), IS3 (94), IS4a (35)
Shigella sonnei Ss046	IS1 (172), IS3 (104), IS4a (32)
Streptococcus suis ST1	IS200 (41)
Xanthomonas oryzae KACC10331	IS1595 (68), IS5a (70), IS5b (80), IS701 (65)
Xanthomonas oryzae MAFF 311018	IS1595 (73), IS5a (83), IS5b (73), IS701 (61)
Xanthomonas oryzae PXO99A	IS1595 (76), IS5a (89), IS701 (95)
Xanthomonas oryzae BLS256	IS1595 (27)
Yersinia pestis A1122	IS200 (66), IS21 (44)
Yersinia pestis Angola	IS200 (99)
Yersinia pestis Antiqua	IS200 (68), IS21 (69)
Yersinia pestis biovar Medievalis str. Harbin 35	IS200 (60)
Yersinia pestis biovar Microtus str. 91001	IS200 (47)
Yersinia pestis CO92	IS200 (64), IS21 (43)
Yersinia pestis D106004	IS200 (58)
Yersinia pestis D182038	IS200 (63)
Yersinia pestis KIM 10	IS200 (52)
Yersinia pestis Nepal516	IS200 (63)
Yersinia pestis Pestoides F	IS200 (54)
Yersinia pestis Z176003	IS200 (62)

Genomes that contain an abnormally high copy number for any IS family. The number in parentheses is the copy number.
